# Nervous Facilitation in Cardiodynamic Response of Exercising Athletes to Superimposed Mental Tasks: Implications in Depressive Disorder

**DOI:** 10.2174/1745017901511010166

**Published:** 2015-09-23

**Authors:** Filippo Tocco, Antonio Crisafulli, Raffaele Milia, Elisabetta Marongiu, Roberto Mura, Silvana Roberto, Francesco Todde, Daniele Concu, Salvatore Melis, Fernanda Velluzzi, Andrea Loviselli, Alberto Concu, Franco Melis

**Affiliations:** 1 Laboratory of Sports Physiology, Department of Medical Sciences, University of Cagliari, Italy; 2 2C Technologies Inc., Academic Spinoff, University of Cagliari, Italy; 3 Obesity Units, Department of Medical Sciences, University of Cagliari, Italy

**Keywords:** Cardiodynamic response, major depressive disorder, nervous motor command, nervous facilitation

## Abstract

Introduction : Motor commands to perform exercise tasks may also induce activation of cardiovascular centres to supply the energy needs of the contracting muscles. Mental stressors per se may also influence cardiovascular homeostasis. We investigated the cardiovascular response of trained runners simultaneously engaged in mental and physical tasks to establish if aerobically trained subjects could develop, differently from untrained ones, nervous facilitation in the brain cardiovascular centre. *Methods :* Cardiovascular responses of 8 male middle-distance runners (MDR), simultaneously engaged in mental (colour-word interference test) and physical (cycle ergometer exercise) tasks, were compared with those of 8 untrained subjects. Heart rate, cardiac (CI) and stroke indexes were assessed by impedance cardiography while arterial blood pressures were assessed with a brachial sphygmomanometer. *Results :* Only in MDR simultaneous engagement in mental and physical tasks induced a significant CI increase which was higher (p<0.05) than that obtained on summing CI values from each task separately performed. *Conclusion :* Aerobic training, when performed together with a mental effort, induced a CI oversupply which allowed a redundant oxygen delivery to satisfy a sudden fuel demand from exercising muscles by utilizing aerobic sources of ATP, thus shifting the anaerobic threshold towards a higher work load. From data of this study it may also be indirectly stated that, in patients with major depressive disorder, the promotion of regular low-intensity exercise together with mental engagement could ameliorate the perceived physical quality of life, thus reducing their heart risk associated with physical stress.

## INTRODUCTION

Nervous motor commands that arise in the brain to perform exercise tasks may interfere with cardiocirculatory homeostasis by cortical and subcortical radiation of stimuli that act as a feedforward mechanism involving parallel activation of motor, respiratory and cardiovascular centres [1]. Concerning the cardiovascular controllers, these central nervous afferents, named central command [2], reach bulbar neurons deputed to modulate heart and vessel activity together with muscle mechano- and metabo-receptor afferents arising from type III and IV nerve endings from exercising muscles, named exercise pressor reflex [3-5]. Central command and exercise pressor reflex, alone or together [6, 7], may induce in brain stem controllers an excitatory output that results in enhanced chronotropic and inotropic activity of the heart from which an increase in blood flow is obtained. 

Again, it is well-known that mental stressors per se may influence cardiovascular homeostasis. The cardiovascular response to mental stress includes a combination of increased rate and force of cardiac contraction, skeletal muscle vasodilation, venoconstriction as well as splanchnic and renal vasoconstriction [8]. Concu *et al.* [9] observed that one minute of mental arithmetic tasks induced in seated men significantly increases in stroke volume (SV) due to augmented inotropic activity, i.e. myocardial contractility increased, together with an increase in chronotropic activity since heart rate (HR) also increased and, as a consequence, cardiac output (CO) increased. Since it is well known that with both haemoglobin concentration and oxyhaemoglobin saturation at a constant blood oxygen delivery (DO_2_) rate depends only on CO, therefore the aforementioned CO increase might result in increased DO_2_. Interestingly, these mental stressors induced cardiodynamic increases that were higher as the difficulty of arithmetic tasks increased. 

However, the cardiodynamic profile resulting from mental stress may appear somewhat different from that due to motor command. Ciuti *et al.* [10] observed that some differences in the complete haemodynamic profile might occur when the purely mental task is compared to a hand-eye task in the same subjects. In fact, in the former condition the CO increase was mainly due to chronotropic adjustments, while in the latter the increase in CO was especially due to haemodynamic changes.

During their training, running athletes are often stressed by continuously having to solve a hard puzzle [11] concerning, for instance, the race conduction and strategy, or being scolded by their coach. In these conditions, there are three neural inputs to cardiovascular centres: a central command irradiated from the motor command; a mechano- or metabo-reflex from exercising muscle, and centrally mediated sympathetic stimulation activated by mental stressors. Therefore these three inputs should interact with each other in a complex way.

It is very likely that if inputs from mental stressors are simultaneously added to the post-synaptic membrane of the neurons in the cardiovascular centres, in addition to the motor command inputs, both of central and reflex origin, then a nervous facilitation (NF) may occur [12] and the resulting CO increase may be redundant with respect to that due to the motor command only, i.e. in this setup a net DO_2 _increase can be expected. 

In these experiments, we tested athletes engaged in races for which the DO_2_ is of strategic relevance, such as those who compete at distances between several hundred and several thousand meters, i.e. the middle distance runners (MDR). We tested their cardiovascular response while athletes were simultaneously engaged in mental and physical tasks, with the aim of establishing if training could develop NF because of both stimuli arising from motor command and mental stressor. A group of untrained subjects were enrolled for the same tasks and acted as control cluster. 

Also, considering the increasing evidence of a beneficial effect of physical exercise on the mood of patients with depressive disorder, we wanted to see if data from MDR might also be applied to these patients.

## MATERIALS AND METHODOLOGY 

### Subjects 

Eight healthy male (23.8 ± 5.40 years, 67.8 ± 11.09 Kg, 171.80 ± 9.23 cm) middle-distance runners trained for distances ranging from 800 to 3000 meters, who were engaged in Italian middle distance standard competitions, took part in this experiment. MDRs were compared to a group of 8 healthy male subjects (CON) not engaged in any competitive activity (21.20 ± 0.44 years, 64.00 ± 12.02 Kg, 165.00 ± 5.83 cm). None of the recruited subjects had a history of cardiac or respiratory disease or was taking any medication at the time of the study. Each volunteer gave written consent to take part in the study after being properly informed of the procedures and risks. Prior of the tests to the subjects were asked to refrain from caffeine and alcohol for 3 h and from the physical activity for 12 h. All experiments were carried out in a temperature-controlled room (room temperature set at 22ºC, relative humidity between 40-50%). The study was performed according to the Declaration of Helsinki. 

### Tests and Instrumentation 

In the experimental protocol, subjects performed a psycho-physiological test (PPT) which consisted of a sequence of mental and physical stressors. The Colour-Word Interference Test (CWIT) was utilized as mental stressor. This test was set by John Ridley Stroop [13] and later improved in the following half century [14]. Briefly, in the CWIT the basic task is to name the ink colour of words, and performance in this condition is compared with performance in naming the ink-colour of coloured words under conditions where word meanings and ink colours mismatch or are incongruent (e.g., the word *red* printed in green ink). CWIT has been shown to be suitable in evoking cardiovascular responses arising from the brain to bulbar cardiovascular controllers [15, 16]. Renaud and Blondin [17] observed that CWIT represents a good task to study the relationship between attention and cardiac activity, and their experiment also provided indications on how the CWIT may act as an efficient laboratory stressor. A computerized version of the CWIT was utilized in our setting [18]. Briefly, several series of 4 coloured words written with a different colour with respect to the meaning appeared in a random order on a computer screen and the delay between words varied randomly from 1 to 2 s. The subjects had to type the colour of the word on selected keys from the keyboard. If the response lasted more than 0.8 s harassment was provoked by an audio signal. 

A cycle-ergometer exercise was utilized as physical stressor (Tunturi EL-1200, Finland). A week before the PPT, each athlete performed a progressive cycle-ergometer test at 60 rpm, with incremental loads of 20 W min^-1^, starting from a baseline of 20 W, until exhaustion (when subjects could no longer follow the exercise pace), and the peak work load (W_peak_) was assessed [19]. During PPT, subjects exercised at a workload that corresponded to 40% of own W_peak_. We chose this low fraction of W_peak_ with the aim of avoiding any kind of pain stressors [20] which, indeed, could interfere with central command if the anaerobic threshold (AT) was exceeded. 

During PPT, cardiodynamic variables were continually and noninvasively measured by using an impedance cardiograph (NCCOM 3, BoMed, Irvine, USA). The impedance method is commonly used to assess haemodynamics in resting, exercising, and recovering subjects [21-25] as well as during mental tasks [26-28]. The device was connected to the subject by applying eight spot electrodes. Dual lower thoracic voltage sensing electrodes were placed laterally to the xiphoid process of the sternum along the mid-axillary line. Two cervical voltage-sensing electrodes were placed as close as possible to the clavicles at the lateral aspect of the base of the neck. The current-injecting electrodes (2.5 mA, 70 KHz) were placed five centimetres above the cervical sensing electrodes and below the thoracic sensing electrodes. An example of recorded tracks of electrocardiogram (ECG) and transthoracic electric bioimpedance (TEB) are presented in Fig. (**1**). Afterwards, we performed the data analysis of recorded traces [29] by using a digital chart recorder (AD Instruments, PowerLab 8sp, Castle Hill, Australia). Stroke volume (SV) was calculated from TEB tracks by employing the following Sramek-Bernstein equation [30]: 

[SV = (VEPT) (Z_0_^-1^) (dZ/dt)_max_ (VET)]

This equation implements the electrical counterparts of the main hydraulic contributors (left ventricle hydraulic capacity, end-diastolic volume, peak ejection velocity, ventricular ejection time) to the left SV. In fact, VEPT is the volume of electrically participating tissues and represents the counterpart of the left ventricle hydraulic capacity. Throughout multiple direct measurements of anatomically normal adults [30], it was observed that VEPT = L^3^·4.25^-1^ = cm^3^, where L (cm) is the distance between the planes on which the thoracic and cervical sensing electrodes lie. Z_0_ (Ohm) is the base impedance of the thorax at the end of diastole, and it is inversely related to thoracic blood volume since decreases in thoracic blood volume increase Z_0_ and vice versa [31], in as much as several findings assumed Z_0_^-1 ^as a reliable index of end diastolic volume both at rest [32, 33] and during exercise [34-36]. dZ/dt_max_ (Ohm·s^-1^) is the maximal Z_0_ first derivative, and it has been shown that dZ/dt_max_ well represents an impedance index of left ventricle ejection velocity [37]. VET (s) is the left ventricle ejection time and is obtained by measuring the time between the onset of the rapid increase in dZ/dt and the negative peak following dZ/dt_max_ [38]. Therefore variables in the foregoing Sramek-Bernstein equation can be quantified as follows:

[SV = (cm^3^) (Ohm^-1^) (s) = cm^3^]

which is the left ventricle ejected blood volume at each beat. 

During PPT both systolic (SBP = mmHg) and diastolic (DBP = mmHg) arterial blood pressures were assessed with a brachial sphygmomanometer. These blood pressure values were assessed at the end of each min.

### Experimental Design

Both CON and MDR groups underwent the PPT which consisted of:

- *Control test *[C] - subjects stayed 4 min at rest while seated on the cycle ergometer;
- *Psychological test* [PsT] – subjects performed a CWIT which lasted 4 min while they rested seated on the cycle ergometer;
- *Physical test* [PhT] – it consisted of a linear increment of workload of 10 W every min, starting from 20 W, at a pedalling frequency of 60 rpm, up to a work load of 40% of W_peak_ which was maintained for 4 min and
- *Psychological and physical test* [PsT&PhT] – while pedalling at a work load of 40% W_peak_ subjects performed the CWIT for the next 4 min.

Each of the PsT, PhT and PsT&PhT tests, preceded by a control test, was randomly performed on different days. To avoid the exercise-induced risk of blood pooling in the leg veins [23] after PhT and PsT&Ph, subjects kept pedalling at a lower work load (10 W·min^-1^) for 4 min, then stopped the exercise. 

### Data Acquisition and Statistics 

During PPT the following variables were considered: HR (beats·min^-1^) which was calculated as the reciprocal of the R-R interval in the ECG trace; stroke index (SI = mL·m^-2^) which was calculated as the ratio between SV and body surface area; cardiac index (CI = mL·min^-1^·m^-2^) which was calculated as the ratio between CO (obtained by multiplying SV·HR) and body surface area; mean arterial blood pressure (MBP = mmHg) which was calculated as DBP + (SBP – DBP)/3. In both groups the cardiodynamic variables obtained in Ps&PhT were compared with those obtained by summing values obtained in PsT and those obtained in PhT (PsT+PhT). 

For data from the PsT, PhT and Pst&PhT tests we chose the use of percent changes with respect to the value in the C test instead of absolute values to describe time courses of variables because we expected that the mild exercise used would cause slight changes in haemodynamics and metabolism. Thus, percent changes would allow the curtailing of inter-individual variance and highlight small perturbations in parameters better than absolute values. 

Repeated measure two-way ANOVA was applied to find significant differences in parameter changes (factors: conditions and times) followed by Newman-Keuls post hoc analysis when significant F-values were obtained. Differences were considered significant if *P* <0.05.

## RESULTS

Table **1** shows mean ± SD haemodynamic variable values in both MDR and CON during task C. Fig. (**2**) shows that during Ps&PhT the HR of both CON and MDR was significantly higher than in PsT+PhT (more than one and a half times). Moreover, MDR showed that HR values during Ps&PhT were also significantly higher than those shown by CON.

**Fig. (3):**
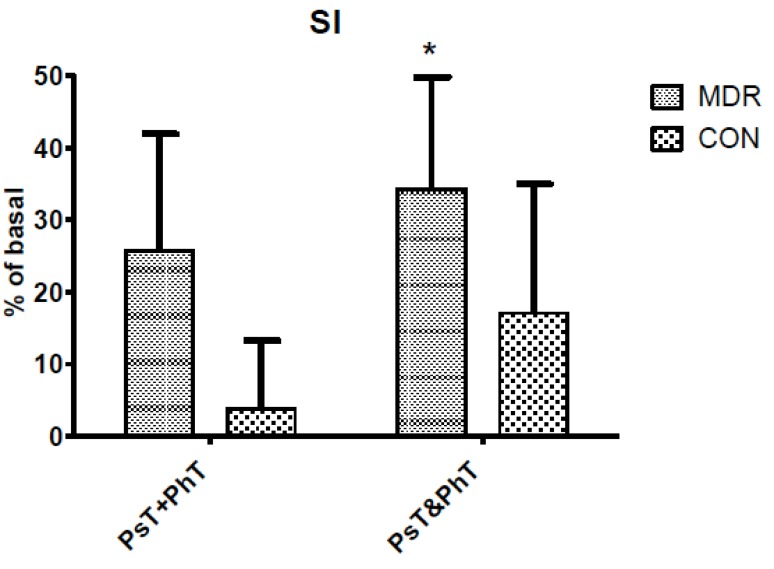
Stroke Index (SI) changes between middle distance runners (MDR) and controls (CON) during conditions Ps+PhT and Ps&PhT. Values are means ± SD percentages of basal. «= *P *< 0.05 *vs. *Ps+PhT.

During Ps&PhT, only MDR showed SI values significantly higher than in PsT+PhT (more than 30%) while in CON it did not. No difference was observed between groups during the latter task (see Fig. **3**).

Differently, in Fig. (**4**) CI values during Ps&PhT were significantly higher in MDR than in CON. It can be noted that while MDR showed a CI value in Ps&PhT, which was significantly higher than in PsT+PhT (about + 80%), the difference was not statistically significant when considering CON.

As concerns MBP, Fig. (**5**) shows no significant modification between Ps&PhT and PsT+PhT in both groups. 

## DISCUSSION

Theoretically, on the basis of classic experiments regarding the laws that govern the nervous reflex centres [39-42], during the Ps&PhT a spatial summation on the post-synaptic membrane (PSM) of the brain stem cardioregulatory neurons of the stimuli coming from the three neural inputs to cardiovascular centres: the central command irradiated from the motor command, the mechano- or metabo-reflex from exercising muscle, and centrally mediated sympathetic stimulation activated by mental stressors, may take place. This post-synaptic potential (PSP) summation could reach a NF in cardiovascular regulatory neurons, for which the excitatory effect of their output on cardiovascular effectors is amplified with respect to what is expected by summing separately each central command, pressor reflex and mental stressor effect. These post-synaptic mechanisms are supported by the observation that in healthy subjects’ interlimb reflexes a NF was recently observed by Nakajima *et al.* [43] during simultaneous arm and leg cycling. 

On the contrary, it may be that this central and reflex PSP summation on PSM of cardiovascular modulating neurons ought to result in a nervous occlusion (NO) [44] for which the excitatory effect of their output on cardiovascular effectors is lower with respect to the effects resulting from central command, pressor reflex and mental stressor summation, separately activated. 

In running athletes, especially those engaged in aerobic races, nervous stimuli from these three sources of information often reach all together the neurons of the cardiovascular modulator centre in the brain stem, resulting either in NF or NO effects. The effects of NF on cardiovascular activity can be quite different from those due to NO. In fact, a facilitation effect originating from cardio-regulating neurons under the central command while the pressor reflex is also engaged (i.e. the motor command), as long as the mental stressors enhances this effect, may give aerobic runners a not negligible boost in reaching a good performance since it could result in an additional CO increase with respect to what is evoked by the motor command alone. The consequent increase in blood outflow from the left ventricle leads to an augmented DO_2 _for a given muscular effort, resulting in a better performance. Obviously, in the case of prevailing NO, since a mental stressor facilitator effect on motor command results inconsistent, none of these advantages can be expected.

In these experiments, when a mental stressor is superimposed on MDR while subjects were exercising, CI increased more than the sum of its separate increases elicited by the mental stressor only and by the physical stressor alone. Although a similar trend was also observed in CON, it did not reach a statistically significant difference. These observations reasonably indicate that subjects trained for aerobic performances rather than untrained ones better developed such an adaptation (presumably housed in their brain stem neurons deputed to modulate cardiovascular activity) which ought to result in a marked post-synaptic facilitation when stimuli arising from the brain, i.e. both motor command and mental stressors, together with stimuli from muscle receptors, induced a spatial summation of PSP on the membrane of those neurons.

Both chronotropic and inotropic heart adjustments concurred to potentiate the CI response induced by psychic and physical stressors when the latter were provided together with the MDR. In fact, both HR and SI showed a redundant increase during Ps&PhT with respect to the increase calculated by summing the two values measured during PsT and PhT. However, HR increased more than SV, and this may mean that the role played by the mental stressor in facilitating PSM of brain stem neurons controlling cardiovascular function is of considerable importance [45].

Interestingly, no significant increase in MBP corresponded to the exceeding response of CI triggered by combined mental and physical tasks. Considering the general law of blood flow for which CI depends on the MBP-to-systemic vascular resistance (SVR) ratio, since MBP had not changed, it is reasonable to hypothesize a SVR decrease. This hypothesis implies that the excess of CI may occur in MDR without increasing cardiac work. Thus, Ps&PhT may have induced in the bulbar controllers of these athletes both cardio-accelerating and vasodilating effects and this consented to avoid supplementary cardiac work while enhancing CI. 

Effects of combined psychological and physical stressors on cardiovascular responses was previously studied by Rousselle *et al.* [46]. In a group of male subjects who performed simultaneously a mental arithmetic task and moderate aerobic exercise (at about 50 W of work load) these authors observed that cardiovascular responses were greater than those during each stressor alone. Previously, similar results had been obtained by Myrtek *et al.* [47], and no different behaviour in cardiovascular response was shown by Siconolfi *et al.* [48] when a mental arithmetic task was performed combined with an exercise at a work load corresponding to 60% of HR peak by coronary artery disease patients. 

Our findings highlight the greater facilitating effect played on cardiovascular activity in fit subjects with respect to controls when mental tasks were combined with exercise. In a previous paper [49] Acevado *et al.* studied the cardiorespiratory responses of low fit and high fit individuals to a mental challenge during exercise at a similar relative intensity. These authors observed that the high fit subjects tended to respond to the dual stress of exercise and mental challenge with a relative increase in HR higher than the control group. Their results agree with ours. 

However, it must be taken into consideration that HR alone is not an exhaustive cardiovascular variable to report the effective pumping activity of the heart, which is much better represented by CO. In the present study the experimental protocol included assessment of CO together with its chronotropic and inotropic conditioners, HR and SV respectively, as well as their derivative haemodynamic variable, i.e. MBP. This more complete cardiovascular profile consents to understanding, better than in previous experiments, how cardiovascular structures disrupt their homeostatic condition in response to either internal or external stimuli. In fact, it was observed that SV rose while a striking fall in HR occurred, due to a post-exertional hypotension [23], in such a way as to maintain normal CO values as long as possible to avoid syncopal asystolia, i.e. in this case HR behaviour alone did not give complete information about heart function. In the same way, during a dynamic incremental exercise HR did not increase because of a complete atrio-ventricular block [50], or during a static exercise while HR was fixed at resting value [51] and thus, in these two experimental settings, CO increased totally by means of SV. On the other hand, it has been observed that HR increments do not always lead to corresponding increases in CO: a similar haemodynamic scenario has been demonstrated when little or no increase in SV takes place, as occurred in the case of untrained subjects while performing an incremental cycle ergometer exercise [52], or when SV reaches a plateau in correspondence to AT during an incremental test, while HR continued to rise as work load was augmented [53].

It has been observed that physical activity appears to be a good adjunctive treatment in the long-term management of major depressive disorder (MDD) patients [54] as well as in improving in these patients the perceived physical quality of life [55]. However, Penninx *et al.* [56] examined the effect of aerobic and resistance training among older persons with depressive symptomatology and they observed that aerobic exercise significantly lowered depressive symptoms over time while no such effect was observed for resistance exercise. 

From the latter considerations and also in considering the results of these experiments, it can be deduced that by subjecting MDD patients to a moderate aerobic training schedule that also implements mental tasks, it may be possible to reach a redundant DO_2_. The latter, in turn, can satisfy sudden fuel demands from exercising muscles by utilizing aerobic sources of ATP, thus shifting AT towards a higher work load, and this may allow these patients to reach a higher aerobic performance at a lower heart cost, i.e. a condition that is conducible to the lowering of cardiac risk [57]. Considering that a recent study by Elderlon and Whooley [58] clearly observed a very close link between MDD and serious cardiovascular disorders, a take-home message from this study may also be that of promoting regular low-intensity exercise together with mental engagement to ameliorate in these patients the perceived physical quality of life, in this way reducing heart risk associated with physical stress.

Borrowing the conclusions of the recent review by Mura and Carta [59], in which they stated that in the last 20 years little progress has been made in showing the efficacy of exercise on depression, we can conclude that this can be charged to the persistent lack of high-quality research as well as clinical issues of management of depression in late life. However, difficulties in establishing the real effectiveness of exercise on depressive symptoms also contributes to this failure. 

In conclusion, concerning possible fall-out effects of these results on MDD clinical treatment, practice of moderate exercise may reduce the cardiac risk in MDD patients when they perform physical activity. 

## Figures and Tables

**Fig. (1):**
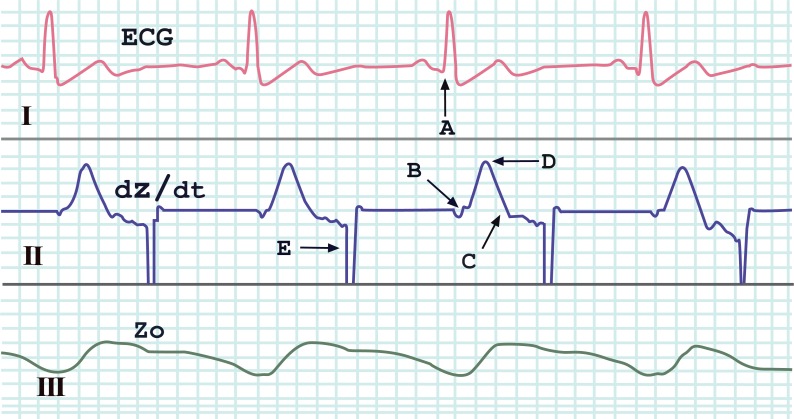
Shown are traces of ECG (channel I), first derivative of Z_0_ (dZ/dt, channel II) and thoracic electrical impedance (Z_0_, channel III), as measured by an impedance cardiograph (NCCOM3). The critical points for determining haemodynamic variables are distinguished: *A* the beginning of the QRS complex, *B* the beginning of the widest deflection of the dZ/dt, *C* the inverse deflection in the dZ/dt trace, *D* the peak of dZ/dt (dZ/dt_max_), *E* automatic reset synchronous with the P wave of the ECG.

**Fig. (2):**
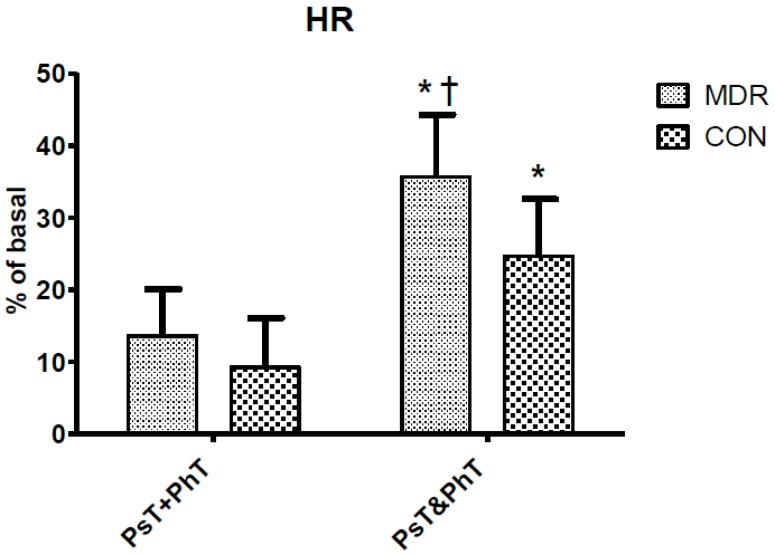
Heart Rate (HR) changes between middle distance runners (MDR) and controls (CON) during conditions Ps+PhT and Ps&PhT. Values are means ± SD percentages of basal. «= *P *< 0.05 *vs. *Ps+PhT. †=* P *< 0.05 *vs. *Ps&PhT of CON.

**Fig. (4):**
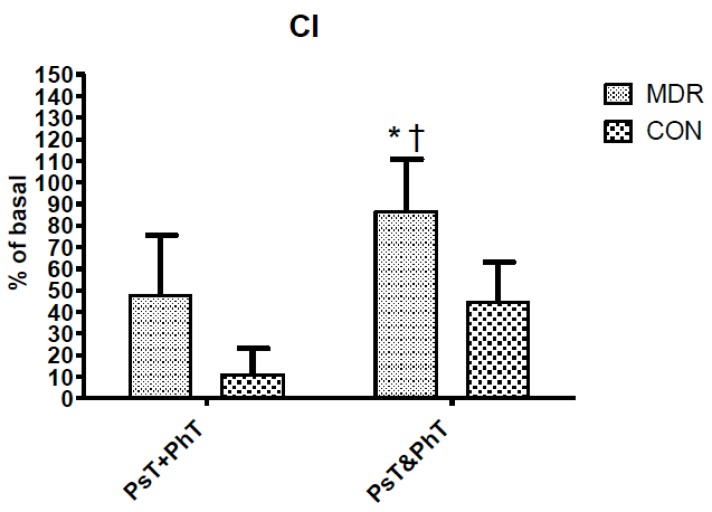
Cardiac Index (CI) changes between middle distance runners (MDR) and controls (CON) during conditions Ps+PhT and Ps&PhT. Values are means ± SD percentages of basal. «= *P *< 0.05 *vs. *Ps+PhT. †=* P *< 0.05 *vs. *Ps&PhT of CON.

**Fig. (5):**
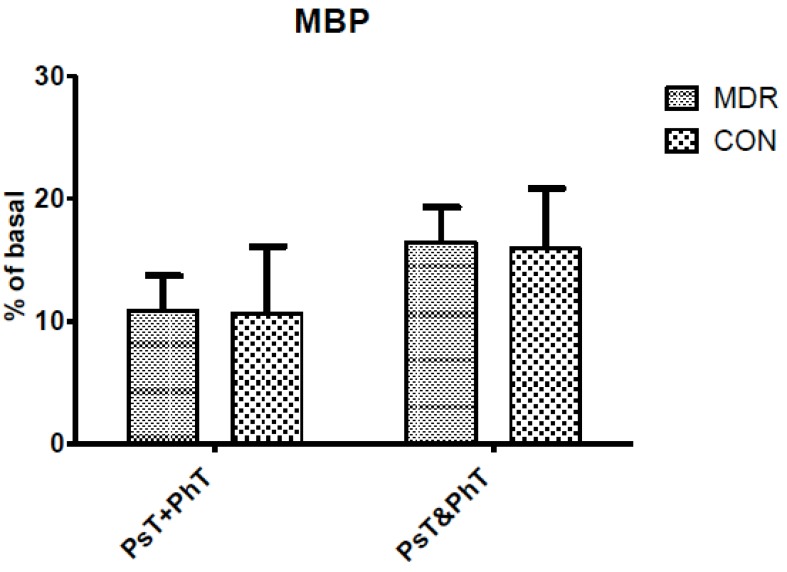
Mean Blood Pressure (MBP) changes between middle distance runners (MDR) and controls (CON) during conditions Ps+PhT and Ps&PhT. Values are means ± SD percentages of basal.

**Table 1.** Mean ± SD values of haemodynamic data during the control test (C) in middle distance runners (MDR) and controls (CON). HR= heart rate; MBP= mean blood pressure; SI= stroke index; CI= cardiac index.

Variable	MDR	CON
HR (beats∙min-1)	78.1±11.0	81.6±19.4 (ns)
MBP (mmHg)	82.3±6.8	77.0±11.0 (ns)
SI (mL∙m-2)	47.8±4.7	41.3±9.4 (ns)
CI (L∙min-¹∙m-2)	3.7±0.7	3.5±1.3 (ns)

## LIMITATION OF THIS STUDY

A possible limitation of the present investigation is the use of impedance cardiography to measure haemodynamic variables. It should be considered that the “gold standard” for haemodynamic assessment includes the Fick and dye-dilution methods, which, however, are invasive and not advisable in subjects who do not require invasive procedures for clinical purposes. Among noninvasive techniques, the choices are restricted to re-breathing, Doppler echocardiography, and impedance cardiography. The impedance method, like re-breathing and Doppler echocardiography, suffers from some limitations [60]. Probably the major source of error with this technique is that chest movements may affect impedance traces by generating artifacts. To overcome this potential source of error, a post acquisition analysis procedure, selected from stored signal impedance traces not affected by artifacts, was used in this study. We previously used the same method in similar studies [61-63]. This method, although hard to follow, has been demonstrated to be reliable and reproducible. Moreover, the mild exercise protocol chosen in the present investigation did not generate either great enhancement in ventilation or marked chest movements. Another possible limitation of this study may arise from the absence of a group of MDD patients in the experimental protocol. Indeed, the idea that the protocol applied to healthy subjects may have important repercussions also for MDD patients occurred to us only after seeing the experimental results. For this reason we believe that the application of this experimental protocol in MDD patients is preferred in the future.

## ABBREVIATIONS

ANOVA: = Analysis of Variance
AT: = Anaerobic Threshold
ATP: = Adenosin Triphosphate
C: = Control test
CI: = Cardiac Index
CO: = Cardiac Output
CON: = Control group
CWIT: = Colour-Word Interference Test
DBP: = Diastolic Blood Pressure
DO_2_: = Blood Oxygen Delivery
dZ/dt_max_: = Maximal Z_0_ First Derivative
ECG: = Electrocardiogram
HR: = Heart Rate
MDR: = Middle Distance Runners Group
MBP: = Mean Blood Pressure
MDD: = Major Depressive Disorder
NF: = Nervous Facilitation
NO: = Nervous Occlusion
PhT: = Physical Test
PPT: = Psycho-Physiological Test
PSM: = Post-Synaptic Membrane
PSP: = Post-Synaptic Potential
PsT: = Psychological Test
R-R: = Time interval between two consecutive R waves in the ECG
SBP: = Systolic Blood pressure
SD: = Standard Deviation
SI: = Stroke Index
SV: = Stroke Volume
SVR: = Systemic Vascular Resistance
TEB: = Transthoracic Electrical Bioimpedance
VEPT: = Volume of the Electrically Participating Tissues
VET: = Ventricular Ejection Time
W: = Work Load
W_peak_: = Peak Work Load
Z_0_: = Base Impedance of the Thorax
